# Recapitulating Tumorigenesis *in vitro*: Opportunities and Challenges of 3D Bioprinting

**DOI:** 10.3389/fbioe.2021.682498

**Published:** 2021-06-22

**Authors:** Gabriela S. Kronemberger, Guilherme A. S. C. Miranda, Renata S. N. Tavares, Bianca Montenegro, Úrsula de A. Kopke, Leandra S. Baptista

**Affiliations:** ^1^Nucleus of Multidisciplinary Research in Biology (Numpex-Bio), Federal University of Rio de Janeiro Xerém, Duque de Caxias, Brazil; ^2^Laboratory of Tissue Bioengineering, National Institute of Metrology, Quality and Technology (Inmetro), Duque de Caxias, Brazil; ^3^Post-graduation Program of Translational Biomedicine (Biotrans), Unigranrio, Duque de Caxias, Brazil; ^4^Post-graduation Program in Biotechnology, National Institute of Metrology, Quality and Technology (Inmetro), Duque de Caxias, Brazil

**Keywords:** tumor microenvironment, tumorigenesis, 3D cell culture, spheroids, organoids, drug assessment, high-throughput screening, 3D bioprinting

## Abstract

Cancer is considered one of the most predominant diseases in the world and one of the principal causes of mortality per year. The cellular and molecular mechanisms involved in the development and establishment of solid tumors can be defined as tumorigenesis. Recent technological advances in the 3D cell culture field have enabled the recapitulation of tumorigenesis *in vitro*, including the complexity of stromal microenvironment. The establishment of these 3D solid tumor models has a crucial role in personalized medicine and drug discovery. Recently, spheroids and organoids are being largely explored as 3D solid tumor models for recreating tumorigenesis *in vitro*. In spheroids, the solid tumor can be recreated from cancer cells, cancer stem cells, stromal and immune cell lineages. Organoids must be derived from tumor biopsies, including cancer and cancer stem cells. Both models are considered as a suitable model for drug assessment and high-throughput screening. The main advantages of 3D bioprinting are its ability to engineer complex and controllable 3D tissue models in a higher resolution. Although 3D bioprinting represents a promising technology, main challenges need to be addressed to improve the results in cancer research. The aim of this review is to explore (1) the principal cell components and extracellular matrix composition of solid tumor microenvironment; (2) the recapitulation of tumorigenesis *in vitro* using spheroids and organoids as 3D culture models; and (3) the opportunities, challenges, and applications of 3D bioprinting in this area.

## Introduction

Cancer remains one of the most predominant diseases in the world in the 21st century, affecting millions of patients per year (Roy and Saikia, 2016). Rather than responding appropriately to signals that maintain cell behavior, cancer cells grow and proliferate without control, invading normal tissues and organs, and eventually spreading throughout the organism (Chambers et al., 2002). The cellular and molecular mechanisms involved in the development and establishment of solid tumors is known as tumorigenesis. It is widely accepted that tumorigenesis is a multistep process, depending on a sequential accumulation of mutations of tissue cells (Ashkenazi et al., 2008). The tumor microenvironment is composed of non-cancerous cells with functions in all stages of tumorigenesis by both stimulating and/or facilitating abnormal cell proliferation (Arneth, 2019).

In recent years, literature has advanced in the better understanding of tumor microenvironment (DeBerardinis, 2020). The non-cancerous cell types include fibroblasts, endothelial cells, and immune cells (Casey et al., 2015; Jarosz-Biej et al., 2019). In addition, depending on the type of tumor, organ-specific interstitial cells are also present. According to previous descriptions, these cells are denominated as “tumor stroma” and, together with the extracellular matrix (ECM), oxygen levels and pH, constitute the tumor microenvironment (Briest et al., 2012; Hirata and Sahai, 2017). This complex interaction between tumor and non-tumor cells leads to an altered metabolism and ECM production. The better understanding of tumor microenvironment is a key challenge to address, contributing to the development of new drugs and treatments (Valkenburg et al., 2018).

In this context, 3D cell culture has gained space in literature due to its advantages compared with “classical” 2D cell culture. 3D cell culture can recreate a sort of tissue microenvironment, providing more accurate data about cell-to-cell interactions, cell-to-extracellular matrix interactions, tumorigenesis, drug discovery, gene expression, metabolic profiling, and protein profiling of the cells. 3D cell culture, such as spheroids and organoids, has the potential to provide alternative models to study tumor microenvironments (Nath and Devi, 2016; Jensen and Teng, 2020). In tumor biology, spheroids are represented by cancer cell lineages self-assembled in rounded shape and organoids by cells derived from tumor biopsies, including cancer stem cells, self-assembled in amorphous shape. Furthermore, cell culture platforms of tumor spheroids and organoids start to be adapted as a model for drug assessment and high-throughput screening (HTS) (Kondo et al., 2019; Heredia-Soto et al., 2020; Renner et al., 2020).

3D bioprinting is a promising emergent bottom–up technology to develop complex tissue models *in vitro*. 3D bioprinting is a form of additive manufacturing, where cells, biomaterials, and soluble factors can be assembled layer by layer (Mandrycky et al., 2016). From 3D bioprinting, it is possible to hierarchically organize tissues, as they are found *in vivo*, and faithfully recapitulate their morphology as well as functional aspects (Datta et al., 2018). Although 3D bioprinting represents a promising technology, main challenges still remain such as the speed of bioprinters and better bioinks for improving cell survival and function in cancer research. The main objective of this review is to explore the cellular and molecular composition of solid tumor microenvironment, the recapitulation of tumorigenesis and drug assessment using spheroids and organoids, and the opportunities and challenges of 3D bioprinting in this field.

## The Tumor Microenvironment

### Background

The tumor microenvironment is heterogeneous, composed mainly of tumor cells and endogenous stromal cells (non-cancerous) that are later recruited by the tumor itself. This microenvironment also contains extracellular components: ECM proteins, extracellular vesicles, cytokines, growth factors, and hormones nourished by a vascular network. The stromal cells are represented by endothelial cells, mesenchymal stem/stromal cells (MSCs), fibroblasts, and macrophages (Wu and Dai, 2017; O’Loghlen, 2018). During tumorigenesis, tumor cells interact greatly and evolve with this surrounding microenvironment, having profound effects on therapeutic efficacy (Bussard et al., 2016).

All tumor microenvironment components communicate continuously with each other mainly by (1) cell-to-cell interactions, (2) cell-to-extracellular matrix interactions, and (3) the network of cytokines, proteins, and chemokines that can favor the immune system or the tumor growth. Thus, any disruption in tumor microenvironment signaling will reflect changes of the balance between immune system and tumor (Hui and Chen, 2015; Merlano et al., 2019).

One of the most crucial factors for tumor microenvironment maintenance and progression to metastasis is the vascular network (Naumov et al., 2008; Quail and Joyce, 2013). Tumor vasculature is characterized as being disorganized and leaky, which is associated with altered endothelial cell adherents junction and tight junction formations, both critical to maintain vascular barrier functions. In addition, tumor cells induce programmed necrosis of endothelial cells, thus, increasing vascular leakiness and tumor cell extravasation and metastasis (Yang and Lin, 2017).

### Cell Components of Tumor Microenvironment

In solid tumors, mesenchymal stem cells and fibroblasts, also named as cancer-associated fibroblasts (CAFs), are the main cellular components of the microenvironment. It is well known that in healthy tissues, fibroblasts support tissue repair and homeostasis; however, CAFs is a heterogeneous population that serves a different function compared with resident fibroblasts (Petrova et al., 2018; Ayan et al., 2020), as suggested by Sugimoto et al. (2006) and Kobayashi et al. (2019). The principal functions of CAFs in the tumor microenvironment are: (1) stimulate tumor cell proliferation by growth factor secretion, (2) modify cancer ECM, which will induce tumor progression and metastasis, and (3) modulate the inflammatory components that facilitate tumor initiation, progression, and metastasis (Servais and Erez, 2013; Raffaghello and Dazzi, 2015). Furthermore, CAFs support endothelial cells to start tumor angiogenesis. Endothelial cells offer nutritional support for tumor growth and development, showing a key role in tumor cell protection from the immune system (Arneth, 2019). Tumor endothelial cells are considered one of the main targets of anti-angiogenic therapy (Hida et al., 2013). A study published by Maishi et al. (2016) showed with two different tumor models that endothelial cells in the tumor microenvironment are able to promote tumor metastasis by direct interaction with tumor cells.

Myo-fibroblasts are specialized fibroblasts, a subpopulation of CAFs, which express the alpha-smooth muscle actin protein and are considered major players in the development of different fibrotic diseases, mainly due to their capacity to remodel the ECM (Yazdani et al., 2017; Ribatti and Tamma, 2019). In tumors, these activated fibroblasts can enhance tumorigenesis, angiogenesis, and metastasis by secreting growth factors and cytokines. Besides fibroblasts and endothelial cells, MSCs are present in the tumor microenvironment as well, interacting with tumor cells via the secretion of growth factors or cytokines, and by transferring mitochondria or microRNAs. Residing in tumors, MSCs form a fibrovascular network by differentiating into smooth muscle cells and vascular pericytes, contributing to vascular network extension (Guo and Deng, 2018). At the beginning of tumorigenesis, MSCs have been shown to drive tumor cells toward an invasive, premetastatic state. However, some studies showed that MSCs can also have an inhibitory effect on tumor growth by reducing cytotoxicity effects, pluripotency, and even by influencing macrophage polarization (Ridge et al., 2017).

Pericytes are multipotent perivascular cells with an established role in vasculature development. Studies have already shown that these cells present immune properties and might serve as a reservoir of MSCs to influence in the *in vivo* regeneration of diverse tissues. Pericytes located in the vessels play a significant role in the homeostasis of these vessels, and when recruited, they change their activation stage to MSCs in order to participate in injury events of the tissue (Meirelles et al., 2013). In addition, pericytes are capable of realizing tumor homing and are considered an important cell component of the tumor microenvironment (Ribeiro and Okamoto, 2015). In cancer, pericytes have been explored because of their capacity to stabilize blood vessel structure and permeability. Due to this, it was discovered that pericytes can affect tumor growth and metastasis positively or negatively. The effects of tumor growth are related to establishing a stable vascular network, which will ensure a proper delivery of nutrients to allow tumor cells maintenance and proliferation. However, these cells can prevent tumor cell dissemination by maintaining the permeability of blood vessels (Barrow and Colonna, 2019). Furthermore, many studies have shown that cancer vessels are characterized by abnormal pericyte population of cells and altered pericytes/endothelial cell interactions, which can effectively contribute to metastasis process and progression of cancers, especially perivascular ones such as glomus tumor, myopericytoma, and solitary fibrous tumor/hemangiopericytoma (Mravic et al., 2014; Chen et al., 2016).

Another cell type whose role is largely explored in tumor microenvironment is the adipocyte. Adipose tissue is composed of adipocytes and non-adipocyte cells, including MSCs from adipose tissue and macrophages. These cells release a variety of molecules that enable them to play a paracrine effect in pathological processes such as breast and ovarian cancer (Robado de Lope et al., 2018).

The macrophage is the most prominent immune cell type in the tumor microenvironment (Arneth, 2019). Macrophages have an active role from early carcinogenesis to tumor progression and metastasis, constituting up to 50% of a tumor mass depending on the type of tumor. Previous studies suggest that after infiltrating tumors, macrophages polarize to a M2 phenotype, take on the functions of tumor growth and angiogenesis, tissue remodeling, and suppression of antitumor immunity (Kim and Bae, 2016). Zhang A. et al. (2017) reported that CAFs promoted M2 polarization of macrophages in pancreatic ductal adenocarcinoma, which enhanced tumor cell growth, migration, and invasion.

Another immune population of cells present in the tumor microenvironment is the natural killer cells (NK). NK cells are large granular lymphocytes that control tumor growth by interaction with tumor cells or because they can affect the function of other innate and adaptative cell populations (Melaiu et al., 2020). Interestingly, NK cells show antitumor activity as they have the efficient and fast capacity to recognize and kill tumor cells. This function is mediated through cell-surface receptors, which examine tissue microenvironments for changes in surface and secretory phenotypes, and then alerts the immune system for the presence of infection or of a malignancy agent. Therefore, this function is largely explored for cancer immunotherapy treatments (Bi and Tian, 2017; Barrow and Colonna, 2019; Zhang et al., 2020). According to Fang et al. (2017), the main approaches used for cancer immunotherapy with NK cells are based on the use of cytokines, as IL-2 and isoforms, antibodies, and the adoptive transfer of *ex vivo* NK cells.

T cells also play important functions in the tumor microenvironment, where it is common to find inhibitory receptors. These can inhibit T cell metabolism and influence T cell signaling, both directly and through release of extracellular vesicles. When isolated from tumors, T cells generally show signs of exhaustion and present distinct metabolic features (Lim et al., 2020). Other immune cells that are present and modulate the tumor microenvironment are granulocytes, such as the mastocytes. Early mastocyte cell infiltration has been reported in human and animal tumors, especially in malignant melanoma, breast, and colorectal cancer (Liu et al., 2011; Komi and Redegeld, 2020). Mastocytes have different functions in the tumor microenvironment such as: (1) modulating tumor biology, by influencing in cell proliferation, survival, angiogenesis, and metastasis; and (2) establishing crosstalk with other tumor-infiltrating cells in the microenvironment (Aponte-López and Muñoz-Cruz, 2020).

Currently, different studies discuss the concept and functions of cancer stem cells (CSC) in tumor microenvironments. These cells, also called stem-like cells or tumor-initiating cells (TICs), were first described in 1994 and are a distinct subpopulation of tumor cells. Recently, this subpopulation of cells has been described as having a unique ability to initiate tumor growth and maintenance. In this context, CSC is considered an important target for cancer immunotherapies (Nassar and Blanpain, 2016; Codd et al., 2018). The quantity of CSC in the tumor microenvironment varies according to the tumor type. These cells can be responsible for preserving tumor heterogeneity by retaining self-renewal and differentiation properties. In addition, CSC also plays a role in innate resistance to cancer therapies, which in turn links to their persistence of the tumor in a specific tissue, which can lead to disease recurrence and metastatic spread (Albini et al., 2015). A study performed by Chen et al. (2014) demonstrated that CAFs enrich CSCs through de-differentiation process and reacquisition of stem cell-like properties in lung cancer. Briefly, the main results showed that CAFs develop a paracrine signaling that induce Nanog expression and promote stemness in cancer niche. What is interesting is that it is possible to discover new therapeutic targets to act in this paracrine signaling of CAFs to CSCs.

### The Extracellular Matrix in Tumor Microenvironment

The ECM contains a diversity of proteins, which influence the cell phenotype of specific tissues due to their biochemical and biophysical properties. The principal ECM proteins secreted by cells in the tumor microenvironment are collagen, fibronectin, laminin, vitronectin, and tenascin (Cheng et al., 2020). It is well known that the ECM is highly dynamic because it is constantly being remodeled and degraded from embryogenesis until maturity. This remodeling is crucial for tissues homeostasis; however, dysregulation of ECM dynamics is common in the development of diseases as cancer (Bonnans et al., 2014; Walker et al., 2018).

In the tumor microenvironment, two main modifications are commonly observed in the ECM: stiffness (rigidity) and degradation. The increase in cross-linking between ECM proteins can cause stiffness (Najafi et al., 2019). The enhancement of tumor ECM stiffness is mainly induced by ECM deposition, remodeling by resident fibroblasts and by the transformed epithelium. In addition, the presence of chemokines and growth factors lead to an inflammation state. The inflammation state induces CAFs activation and their transdifferentiation into myofibroblasts, causing tissue desmoplasia. Then, myofibroblasts deposit ECM proteins, secrete growth factors, and apply contraction forces on the tumor ECM. In the end, newly deposited ECM proteins will generate larger and rigid fibers that turn the ECM rigid (Frantz et al., 2010). However, the disruption in the signaling between these ECM proteins will result in degradation, mainly caused by the activation of metalloproteinases (MMPs) (Najafi et al., 2019). The MMPs cleave collagen fibers of tumor ECM and reorganize them into tube-like structures to facilitate cell migration in the microenvironment (Malik et al., 2015).

The MMP genes were previously associated with increased risk and evolution of breast cancer. In the study developed by Slattery et al. (2013), the genetic variation of MMP1 (nine SNPs), MMP2 (eight SNPs), MMP3 (four SNPs), and MMP9 (three SNPs) together with breast cancer risk was evaluated in Hispanic and Non-Hispanic women. The results showed that MMPs have associations with breast cancer progression and prognosis. Overall, MMP-2 showed the strongest gene association with breast cancer development.

Regarding ECM modifications in breast cancer, another study, published by Boghaert et al. (2012) showed, with a 3D cell culture model, that the regions where the tumor cells invaded the breast tissue more was directly correlated with a higher mechanical stress of the host epithelial tissue. The use of a 3D cell culture model to recapitulate the breast tumor microenvironment can then aid in the better understanding of *in vivo* mechanisms.

One of the first studies published correlating abnormal ECM and the progression of cancer was performed by Neglia et al. (1991), which investigated the risk of cancer in patients with cystic fibrosis. The study was developed with North American and European patients with cystic fibrosis, and the results showed that, in fact, these patients had an increased risk to develop digestive tract cancers. In cancer, the abnormal ECM affects the progression of the disease by promoting changes in host cells normal functions. In addition, ECM anomalies are also capable of (1) deregulating the behavior of stromal cells, (2) promoting angiogenesis and inflammation associated with the tumor, (3) leading to the generation and maintenance of an established tumorigenic microenvironment, and (4) can also induce metastatic dissemination (Lu et al., 2012; Seager et al., 2017).

Not only cancer cells but also CAFs lead the modification and remodeling of the ECM during cancer progression. The biochemical cross talk between the cancer cells and CAFs, and the biomechanical changes of the ECM are major contributors to tumor cell migration and invasion, which will influence tumor progression to metastatic state. Additionally, growth factors, chemokines, and metabolic changes released from the ECM contribute to the maintenance and progression of the tumor microenvironment (Erdogan and Webb, 2017; Eble and Niland, 2019).

Due to the importance of ECM modification in the tumor microenvironment, studies are being conducted in order to develop therapeutic treatments to target the cancer ECM. Van der Steen et al. (2017) explored the functionalization of drug-loaded lyophilisomes (albumin-based biocapsules) loaded with doxorubicin and functionalized with antibodies, to act in the ECM, or stroma, of ovarian carcinomas, in order to evaluate its potential to eliminate cancer cells. The principal results showed that drug-loaded lyophilisomes were effective to induce cancer cell death and can be considered as a therapeutic agent to specifically target ECM components of the tumors. In addition, Zhang et al. (2018) explored the use of cyclopamine, a special inhibitor of the hedgehog-signaling pathway, which contributes to ECM formation of pancreatic ductal adenocarcinoma, to ameliorate solid stress and improve nanomedicine delivery to tumor site. The principal results showed that the drug was able to disrupt ECM in pancreatic ductal adenocarcinoma, reduced solid stress of the tumor together with an improvement of function of tumor vessels, which allowed a better perfusion in the tumor area.

Although the drugs discovered recently to target tumor ECM might effectively reduce the number of cancer cells and reduce solid stress, there are still many challenges that ECM components in tumor microenvironment can set that could interfere with therapeutic treatments. Briefly: (1) ECM proteins act as a physical barrier, which makes drug delivery more difficult, (2) ECM proteins can de-differentiate non-CSCs into CSCs, and this can make it harder for the elimination process of CSCs in the microenvironment, (3) the ability of ECM to modulate immune responses, and (4) complex nature of ECM, with its different molecules and isoforms (Nallanthighal et al., 2019). Therefore, the ECM in the tumor microenvironment has a considerable impact in cancer progression and further metastasis. Due to this, a better understanding of the interactions between cancer cells and ECM is needed and might only be addressed by 3D cell culture models, especially in order to have more faith in the results of drug screening to target cancer (Drost and Clevers, 2018).

## 3D Models Recapitulating the Tumorigenesis *in vitro*

### Background

The tumorigenesis of cancer disease is heterogeneous in growth rate, invasiveness, drug sensibility, and individual patient derived characteristics (McGranahan and Swanton, 2017; Fan et al., 2019). Therefore, the *in vitro* and *in vivo* preclinical studies fail in emulating the microenvironment of the tumor to predict its sensibility or its resistance to drugs, or the metabolic and molecular pathways. This explains the low success rate of drug acceptance for oncologic drugs at 3.4% (Wong et al., 2019).

Immortalized cell lines are a valuable resource to investigate the physiological mechanisms and body–environmental interactions between healthy cells and cancerous cells due to their ease of growing and manipulating *in vitro*. Monolayer assays employing immortalized cancer cells are characterized by low cost, less complexity, and are readily employed in the HTS of drug trials and molecular biomarkers (Fan et al., 2019). However, because of the fast proliferation of the monolayers, it is likely that the culture might be affected by problems such as de-differentiation or abnormal gene expression profiles, which may influence the result of experiments as well as be contrasting to *in vivo* tests (Shah et al., 2018). Furthermore, monolayer assays glean so little about the gene expression, reorganization, and responses involved in the tumorigenesis, mainly due the absence of a tumor microenvironment (Gao and Chen, 2015).

To fill the gap between these insufficient or inappropriate models, 3D cultures arise as an urgent tool to improve the prediction system and mechanism of understanding tumorigenesis in humans. 3D cultures allow for systematic investigation into the several unidentified metabolic pathways and cascades (Sawant et al., 2016).

The classical scaffold-based approach in tissue engineering has focused on devising cells, bioactive factors, and scaffolds with biocompatible biomaterials to produce models able to maintain the tumor phenotype (Molina et al., 2020). In these models, it is possible to co-cultivate epithelial and stromal cells and observe the crosstalk of multiple cell types interacting, which regulate normal and neoplastic development (Sawant et al., 2016).

In contrast to scaffold-based methods, scaffold-free approaches emerge as 3D tumor models. The scaffold-free approaches are aggregates of cells, producing several common features that are similar to the solid tumor *in vivo* such as cellular heterogeneity, cell-cell signaling, hypoxia, membrane protein distribution, and gene expression patterns (Zhao et al., 2019).

### Tumor Spheroids

The development of 3D models such as spheroids made it possible to engineer several cancer-like microenvironments *in vitro*. Many papers claim to have developed their protocols to build tumors such as glioblastomas, colorectal, breast, liver, lungs, among others (Kelm et al., 2003; Hirschhaeuser et al., 2010; Chimenti et al., 2017; Eilenberger et al., 2018; Froehlich et al., 2018; Oraiopoulou et al., 2019; Foglietta et al., 2020; Lee et al., 2020).

The breast cell line MCF-7 is an adenocarcinoma-luminal subtype one. The cell morphology is epithelium-like resulting in their ability to self-aggregate into a steady shape, which makes it easier to maintain their viability and to use it for implantation in mice for *in vivo* studies (Do Amaral et al., 2011; Comşa et al., 2015; Froehlich et al., 2018).

HEPG-2 is an epithelial-like hepatocellular carcinoma that, due to the liver cells’ role of the metabolism, is considered a valuable option to study cell genotoxicity (Luckert et al., 2017; Shah et al., 2018). 3D models using HEPG-2 can be used alone in drug screening or as a co-culture with other tumor cell lines (Lan et al., 2010; Jung et al., 2017).

Some aspects must be considered when working with spheroids. One of them is the quality of the 3D protocol, which is related to some variables such as the kind of support for the culture, the non-adherent medium used, the number of cells that are seeded, the spheroid formation technique, the temperature, and the amount of CO_2_ and O_2_ available (Mironov et al., 2009; Mehta et al., 2012; Däster et al., 2017). All these factors are highly changeable according to the tumor line chosen.

The role of hypoxia and the capacity of a tumor to induce neovascularization in its microenvironment using spheroid models have been debated since the early 1990s. It has been established that genetic changes can cause an “angiogenic switch” as the newly mutated cells acquire the ability to upregulate the production of angiogenic factors in comparison to healthy cells, especially in hypoxic niches (Shweiki et al., 1995; Catalano et al., 2013).

Studies using colorectal spheroids and the 5-Fluorouracil drug have indicated that hypoxia and necrosis induction is associated with tumor progression and cell resistance to chemotherapy treatments. The difference in the spheroid size is a variation that also shows its importance in determining whether the mentioned effects moderately or intensely impact the aggressiveness of the tumor (Karlsson et al., 2012; Däster et al., 2017). On the contrary, other studies developed with multicellular spheroids also have demonstrated that when hypoxia–reoxygenation is induced, the levels of vascular endothelial growth factor (VEGF) are downregulated by the tumor cells, as well as it activated DNA damage repair markers (Kondoh et al., 2013; Riffle et al., 2017).

Nevertheless, managing these elements and controlling the long-term viability of the spheroids is an arduous task due to their natural propensity of apoptosis, as a result of poor gaseous and nutrients diffusion (Zhang W. et al., 2016). One possible solution is to use microfluidic systems to allow continued flow of the molecules needed for the spheroids to keep metabolizing and proliferating (Moshksayan et al., 2018). Human lung adenocarcinoma A549 cells, for instance, can be seeded with human endothelial cells in a collagen-I–Matrigel microfluidic device containing a micro-pump to supply the system with oxygen and nutrients. It is a useful protocol for further respiratory system cancer studies (Lee et al., 2019).

Colorectal tumors are likely to be formed at elderly ages, especially over 50 years. It is also the third cause of death among men and women in the United States (Siegel et al., 2020). The communication promoted by cells in the spheroid allows studies to explore the interactions between drugs and the 3D model (Elliott and Yuan, 2011). Concerning this approach, it was shown through spheroid models that the anticancer drug KP1339 triggers an immune cell death *in vitro*, which matches arrays that showed preclinical activity *in vivo* (Wernitznig et al., 2019).

Coming up with a model that mimics the microenvironment of mammary tissue requires a complex mixture of several cell types and tissues, as well as functional ECM and long-term sustainable cell–cell and cell–ECM interactions. In this regard, adipose tissue might work well when co-cultured with mammary cell lines (Kim et al., 2004; Picollet-D’hahan et al., 2016). As a complex tissue, adipose is constituted of several populations of cells such as adipocytes, MSCs, endothelial progenitor cells, pre-adipocytes, lymphocytes, pericytes, and macrophages (Schäffler and Büchler, 2007; Hu and Polyak, 2008).

Studies with co-culture between MCF7 line and MSCs have shown that this mesenchymal population can improve tumor aggressiveness *in vivo* in comparison with MCF7 culture alone. Similar to immune cells, MSCs demonstrate tropism for spots consisting of damaged tissue including tumor microenvironmental sites, cooperating with migration and metastasis (Koellensperger et al., 2017; Chen et al., 2019).

### Tumor Organoids

Different from spheroids, tumor organoids must be derived from human tumor biopsies (Drost and Clevers, 2018; Wang et al., 2020). The advantages and applications of tumor organoids are related to the tissue-specific mutagenic processes accumulating specific types of somatic mutations during malignant transformation in patients. Single stem cell-derived and long-term-cultured organoids were used to determine the genome-wide mutation patterns in distinct healthy stem cells (Wang et al., 2020).

The ability to grow organoids with high efficiency from healthy human adult stem cells has paved the way to grow tumor tissue patient-derived organoids (PDO). So far, long-term organoid cultures have been established from primary colon, esophagus, pancreas, stomach, liver, endometrium, and breast cancer tissues, as well as from metastatic colon, prostate, and breast cancer biopsy samples (Drost and Clevers, 2018).

Another 3D model is the cultivation and testing of the patient-derived tumor xenografts (PDTX) generated in animal models. PDTX is about the implantation of small pieces of tumors from human biopsies into highly immunodeficient mice. After tumor growth, the tumor is transferred into secondary recipient mice. PDTXs often maintain the structures of the original tumors at molecular, cellular, and tissue levels (Drost and Clevers, 2018). Thus, it is able to recapitulate the heterogeneity of the tumor and its native microenvironment; however, it is more incompatible to HTS due to its expensive, time consuming and complex procedure (Hidalgo et al., 2014). Besides PDTX, it is also possible to induce the tumor directly into animal models. However, animals present great phylogenetic distance to humans, have different metabolism, size, and lifespan, which all misdirect the drug development during human clinical trials (Wang, 2019).

The generation of cancer spheroids and organoids, like PDO are low cost, fast compared with PDTX, can be adapted to HTS and allow investigation of the alterations occurring during the initiation and progression of tumorigenesis (Fatehullah et al., 2016; Fan et al., 2019). This is one of the reasons why tumor organoids have been increasingly used as a faithful *in vitro* model system to study cancer metastasis (Fan et al., 2019).

Tumor organoids keep the main pathophysiological features required to identify the critical factors in the acquisition of cancer metastatic potential, which may elucidate mechanisms involved in the metastasis cascade (Fan et al., 2019). On the other hand, one of the intrinsic limitations is the lack of stroma, blood vessels, and immune cells in cultured organoids, especially the immune cells (Wang et al., 2020) due to their regulatory roles in epithelial cell growth and differentiation, invasion, and metastasis (Mueller and Fusenig, 2002; Sawant et al., 2016).

One very interesting strategy when studying tumor organoids is to associate healthy organoids with tumor ones in a fluidic platform called organ-on-a-chip aiming to study metastasis via the circulatory system. These devices mentioned before are microfabricated to emulate a precise microenvironment, controlled, with continuous flow perfusion culture, and high-throughput format (Fan et al., 2019). Jeon et al. (2015) studied 3D vascularized organotypic microfluidic assays to study breast cancer cell extravasation, while Xu et al. (2016) projected a four-organ chip to assess lung cancer metastasis. Huang et al. (2009) found out that the laminar flow properties of microfluidic devices have been leveraged to compartmentalize human mammary fibroblasts in an ECM gel side-by-side with another ECM gel containing breast ductal carcinoma *in situ* cells; this setup revealed that the fibroblasts had to be in contact with the tumor cells to induce the transition to the invasive phenotype (Huang et al., 2009; Benam et al., 2015).

### High-Throughput Screening and 3D Models

Pre-clinical studies fail around 85% in the oncological drug trials, not demonstrating sufficient safety or efficacy (Gao et al., 2015). To overcome this issue, the approaches that enable high-throughput (thousands of cells per experiment) are best suited to efficiently sample the complex cellular diversity in organoids and to understand organoid-to-organoid variability (Brazovskaja et al., 2019).

High-throughput screening provides a practical method to investigate large numbers of pharmaceutical compounds in *in vitro* monolayers assays, being a universal assay in pharmaceutical and Biotech industries (Pereira and Williams, 2007). It has also spawned a billion-dollar industry that supports the increasing demands for speed, capacity, and cost-effective screening of vast libraries of compounds (Pereira and Williams, 2007). The accessibility of HTS data merged with the ToxCast^TM^/Tox21 databases allows for elucidative toxicological considerations seen below (Suh et al., 2018).

**FIGURE 1:**
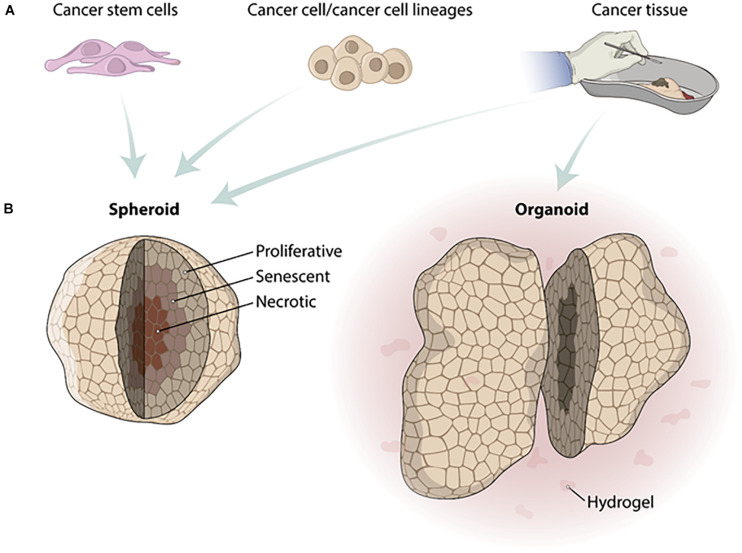
Differences in fabrication of tumor spheroids and organoids. **(A)** Cell types used to produce tumor spheroids and organoids. Spheroids can be fabricated from cancer stem cells, cancer cells/cancer cell lineages, or cancer tissue. Organoids must be fabricated from human cancer biopsies. **(B)** After the fabrication process, tumor spheroids show different zones because of the distinct gradient concentrations of O_2_ and CO_2_. The zones from spheroids inside out are necrotic, senescent, and proliferative. Organoids are usually produced in a hydrogel substrate and do not present a homogeneous size and shape.

Based on the advantages of tissue engineering scaffold-free approaches in recapitulating the tumor microenvironment, mainly represented by spheroids and organoids, a paradigm shift in HTS placing them at the forefront of drug discovery (Li et al., 2016) together with the need to adapt the protocols for the HTS. Spheroids have been adapted for use with several HTS technologies. On the other hand, organoids represent a challenge, mainly due to the presence of hydrogels and their heterogeneity of shape (Figure 1). Furthermore, the most common read-out of HTS technologies is still based on imaging systems making spheroids and organoid depths and their associated light scattering a technical challenge (Li et al., 2016).

So far, tumor spheroids, tumor organoids, and PDTXs are allowed for testing of multiple individual drugs prior to *in vivo* analysis (Beshiri et al., 2018). Gao et al. (2015) established ∼1,000 PDXs with a diverse set of driver mutations against 62 treatments across six indications. Mateo et al. (2015) showed the presence of a homologous recombination deficiency genotype in Metastatic castrate-resistant prostate cancer and predicted responsiveness to Olaparib, which is the first genomic biomarker-driven therapy on track for FDA approval. Another example is for human kidney organoids, where Czerniecki et al. (2018) produced automated organoids and assessed drug effects by HTS.

Liu et al. (2020) review that Kita et al. (2019) screened 2,098 compounds in bladder cancer organoid cell lines. They also discovered that Disulfiram, an anti-alcoholism drug, and cisplatin had a cooperative effect. Lampis et al. (2018), after screening 484 compounds in six cholangiocarcinoma’s organoid cell lines, presented that the sensitivity of HSP90 inhibitors was related to the mutation of MIR21 gene. Kondo and Inoue (2019) reported an advanced system for the HTS of 2,427 drugs using the cancer tissue-originated spheroid; those lines exhibited diverse sensitivities to the hit compounds, demonstrating the usefulness of this system for investigating highly heterogeneous disease.

There is now increasing evidence that the tumor microenvironment affects the efficacy of drugs on the cancer cells (Lal-Nag et al., 2017). Several complex ovarian cancer models have already been published, such as the 3D omental mesothelium model and models that include microfluidics, which demonstrates this (Watters et al., 2018). Currently, the mesothelium model is the only 3D organotypic microenvironment model of ovarian cancer that is used by multiple research groups (Kenny et al., 2007). The mesothelium model recapitulates the main physiological aspects of ovarian cancer cells in the mesothelium lining (Watters et al., 2018). Lal-Nag et al. (2017) proved that several classes of targets were more efficacious in cancer cells growing in the absence of the metastatic microenvironment, and other target classes were less efficacious in cancer cells in pre-formed spheres compared with forming spheroids cultures. These methods were adapted to HTS and to more than 100,000 small-molecule compounds that can potentially identify novel treatments (Watters et al., 2018). Hasan and group reported the use of bioprinting for *in vitro* ovarian cancer tissue modeling for research applicable to HTS. Human ovarian cancer was printed on Matrigel^TM^ to form multicellular acini (Hasan et al., 2011). This approach allows for physiologically relevant cell fabrications and can also provide an alternative to animal testing (Matai et al., 2020).

### Tumor Organoids and Personalized Medicine

As explained in the sections before, tumor organoids must be derived from human biopsies. This outstanding characteristic from tumor organoids has given rise to the creation of tumor biobanks highlighting the concept of personalized medicine to predict effective drugs before the start of the treatment. One crucial challenge to be addressed related to drug testing for cancer models is that the majority of drugs show intratumor heterogeneity, while others are uniformly toxic in all cases. Furthermore, as organoids can be produced from a patient’s own cells, the genetic analyses and drug screening results will be specific to the patient’s tumor (Tellez-Gabriel et al., 2018; Kondo et al., 2019). Some examples are described below.

An organoid biobank of breast cancer tissues from >100 patients was established (Sachs et al., 2018). These organoids represented genetic and histopathological features of breast cancer and maintained the expression of breast cancer biomarkers. This means organoid biobanks have predictive value for drug efficacy in the treatment of individual patients (Wang, 2019), allowing personalized cancer treatment.

Van de Wetering et al. (2015) established tumor organoid cultures from 20 consecutive colorectal carcinoma patients. The results showed that organoids were able to resemble the original tumor characteristics, and gene expression analysis indicated that the majority of consecutive colorectal carcinoma molecular subtypes were properly represented.

Sachs et al. (2018) described a protocol to produce a biobank of human mammary epithelial organoids. The organoids were able to recapitulate the diversity of the disease. Additionally, histological, hormonal, and gene expression analysis resembled the status of the original tumor. Furthermore, the organoids allowed proper drug screening tests when compared with *in vivo* xeno-transplantations.

Yan et al. (2017) developed a primary gastric cancer organoid biobank that comprises normal, dysplastic, cancer, and lymph node metastases from 34 patients. The results showed that organoids were able to closely mimic the morphology, transcriptome, and gene expression profiles when compared with *in vivo* original tumors. It was also seen that organoids were sensitive to unexpected drugs (recently approved or in clinical trials) after drug screening tests.

## 3D Bioprinting

### Background

As discussed previously, 3D cell culture models as spheroids and organoids are capable of better mimicking the tumor microenvironment that is found *in vivo* by recapitulating cellular and molecular events. However, spheroids and organoids follow a non-guided spontaneous formation of tissues and organs by self-assembly mechanism. In this context, because of the ability to precisely guide and organize the position of different cell types and growth factors and also perfusable networks, 3D bioprinting has a potential to improve current models and guide recapitulation of the tumor microenvironment (Datta et al., 2020). The ability to engineer controllable cancer tissue models in high resolution can considerably accelerate cancer research and improve personalized medicine, improving the treatment and life expectancy of cancer patients in the future (Knowlton et al., 2015; Belgodere et al., 2018; Langer et al., 2019).

3D bioprinting is one of the most widely used technologies in tissue engineering and regenerative medicine to develop complex tissues and organs that mimic their native microenvironment (Murphy and Atala, 2014; Moroni et al., 2018). As 3D bioprinting is a process where bioinks, usually composed of hydrogels, and cells are turned into functional tissue-engineered constructs from digital models, it is constantly showing more advantages compared with classical scaffold-based tissue engineering. One of the principal aims of using 3D bioprinting techniques so far is to biofabricate vascular structures (Vijayavenkataraman et al., 2018). This technique integrates biomaterials, living cells, and automated controlled systems to create complex microstructures and precise control over the structures developed compared with other currently available methods (Mandrycky et al., 2016).

Usually, 3D bioprinting begins with a computer-assisted process in order to deposit biologically relevant biomaterials, growth factors, and living cells to generate a desired tissue or organ model. Basically, it is possible to divide the 3D bioprinting process in three: (1) pre-processing for acquiring the 3D computer-aided design (CAD) model of the tissue to be bioprinted, (2) automated deposition of cells, spheroids, biomaterials, or other biological component of interest, and (3) maturation of the tissue constructs (Zhang Y. S. et al., 2017; Datta et al., 2018).

The principal 3D bioprinting techniques are (1) inkjet, (2) extrusion-based, and (3) laser-assisted bioprinting (Huang et al., 2017). In inkjet bioprinting, it is possible to precisely control both the size of the desired tissue pattern, as well as the generated droplets. In this way, it is possible to determine the volume, size, and quantity of a sample to be bioprinted. In terms of precision, it is possible to control the number of the cells per droplet, which is an advantage when scaffolds are being used (Zhang and Zhang, 2015).

Extrusion-based bioprinting is the most used technique that uses the principles of a fluid-dispensing system with a robotic one for extruding materials, which can then be applied to different 3D bioprinting approaches. The fluid-dispensing system can be directed by pneumatic, mechanical, or solenoid forces. Through extrusion-based bioprinting, it is possible to precisely deposit cells, which can be encapsulated in a pre-established design of geometrical filaments and then bioprinted (Ozbolat and Hospodiuk, 2016). However, one of the biggest challenges of this technique is the resolution level that can be reached (Ning and Chen, 2017).

Laser-assisted bioprinting is based on the laser-induced forward transfer (LIFT) principle and is considered a “direct-write” method, which can precisely control the virtual deposition of cells, growth factors, and biomaterial containing droplets at a MHz range speed. Therefore, with this technique, it is possible to achieve high resolution (Devillard et al., 2014). However, the principal disadvantage is the use of the laser directly on the cells, which can damage cell viability (Derakhshanfar et al., 2018).

In order to authentically develop the desired tissue construct, the hydrogel choice is crucial, mainly because the hydrogel will provide the physical and biochemical properties to guide cell proliferation, differentiation, and the final maturation of the engineered construct. In this way, the hydrogel must contain similar properties of the desired tissue when *in vivo* (Gopinathan and Noh, 2018). Several hydrogel formulations have been developed, such as decellularized ECM, alginate, gelatin, hyaluronic acid, and polymers (such as methacrylated gelatin, polyethylene glycol and poly lactic acid) to serve as functional bioinks (Parak et al., 2019).

### Scaffold-Free 3D Bioprinting

The use of bioinks is the foundation of bioprinting. This approach is based on cells and/or biomaterials with specific formulations for each type of cell (Hospodiuk et al., 2017). The ideal formulation of bioinks should meet each cell type’s biological requirements without toxicity to the cells (Gungor-Ozkerim et al., 2018). Their desired properties include printing, mechanical properties, biodegradation, and post-bioprinter maturation (Hong et al., 2018). These properties depend on different parameters such as solution viscosity, surface tension of the bioink, the ability to interconnect on its own, and the properties of the printer nozzle surface. The living cells encapsulated in the bioink grow and occupy the space to form predefined tissue structures (Huang et al., 2017; Gungor-Ozkerim et al., 2018).

However, an important limitation of this approach is that, although cells can be manipulated individually, they do not form mechanically stable assemblies in many cases unless intercellular adhesions are made very strong, possibly by chemical means, which is not ideal for mimicking the tissue microenvironment (Goulart et al., 2019). Additional structural cohesion needs to be produced by the cells, like their own secreted ECM. However, this is a long-term process and depends on the cell type and the ECM deposition quality (Ong et al., 2018; Heo et al., 2020).

The alternative approach of using cells with a pre-assembly of spheroids has been widely studied, as it improves the production capacity of its ECM, in addition to providing greater biomechanical cohesion in larger-scale constructs for bioprinting (Swaminathan et al., 2019). Also, spheroid-based methods are generally milder and, therefore, induce much less or no cell damage during bioprinting (De Moor et al., 2018). Another attractive feature of spheroid bioprinting is its efficiency, as the speed of bioprinting can be increased using large building blocks such as spheroids (Gutzweiler et al., 2017).

An alternative method, still considered to be scaffold-free, can provide temporary support to the spheroids and, thus, facilitate their fusion and maturation in tissue models using a set of microneedles (”Kenzan”). The Kenzan bioprinting method provides a high-resolution biofabrication process, facilitating the fusion of spheroids into larger tissue constructions in a needle matrix removed after spheroid fusion. This method is used in the Bio-3D Regenova Printer marketed by Cyfuse Biomedical (Moldovan, 2018; Murata et al., 2020).

Recently, bioinks were developed using formulations composed only of spheroids with several thousand cells. Studies have shown that spheroids can form tissue threads up to 8 cm in length with rapid spheroid fusion without using aggressive chemicals as crosslinkers or as support materials (Bakirci et al., 2017; Ji and Guvendiren, 2017; Osidak et al., 2019). The spheroid bioinks showed better results than the individual cells because they preserved the integrity of the ECM. The use of bioinks without structure, composed only of cells, has been attracting more and more attention as a bioprinting method for the 3D construction of complex tissues, through which the application of ball-beading constructions is widely addressed (Skardal et al., 2016).

Several spheroid bioprinting techniques have been reported in the literature. One of the first techniques widely explored was extrusion-based bioprinting, in which the spheroids were loaded into a syringe cylinder and extruded into a controlled distribution gel medium. However, the spheroid tips easily deform in the syringe and are subject to breakage during the extrusion process. Simultaneously, the support structures need to be printed in 3D to facilitate the aggregation of extruded ball tips (Mandrycky et al., 2016). A significant advance was made using the Kenzan method. However, the method has limitations inherent to the accuracy of the 3D bioprinting process (Moldovan et al., 2017). To overcome some of the greatest challenges of the current techniques, recent studies have shown that aspiration-assisted bioprinting allows accurate bioprinting of spheroids over sacrificial or functional gel substrates (Chimene et al., 2016). In a mold of sacrificial material, such as alginate or agarose, the material is discarded as the bioprinted tissue matures and subsequently deposits its components in the ECM (Ayan et al., 2020).

Although methods using sacrificial gel substrates do not present the common problems of inkjet and microextrusion (such as nozzle clogging), they still have their technical limitations (Vijayavenkataraman et al., 2018; Adhikari et al., 2021). One of these limitations is the time necessary to form and maturate spheroids prior to bioprinting. In addition, the development of specific bioinks compatible with the characteristics of most spheroid types is essential for the viability and correct maturation of each tissue (Li et al., 2016).

Large-scale production is also still a great challenge (Datta et al., 2020). Studies on standardization and automation of spheroid production are essential for building more complex and genuine-sized tissues in the future. Moreover, an important implication in biofabrication is training for the unique skills and techniques required of this technology’s users and operators. Finally, the bioprinting of 3D cell constructs originating from spheroids composed of various types of cells has been studied to increase the functionality of these 3D constructs (Sasmal et al., 2018; Swaminathan et al., 2019).

### 3D Bioprinting of Tumor Models

**FIGURE 2:**
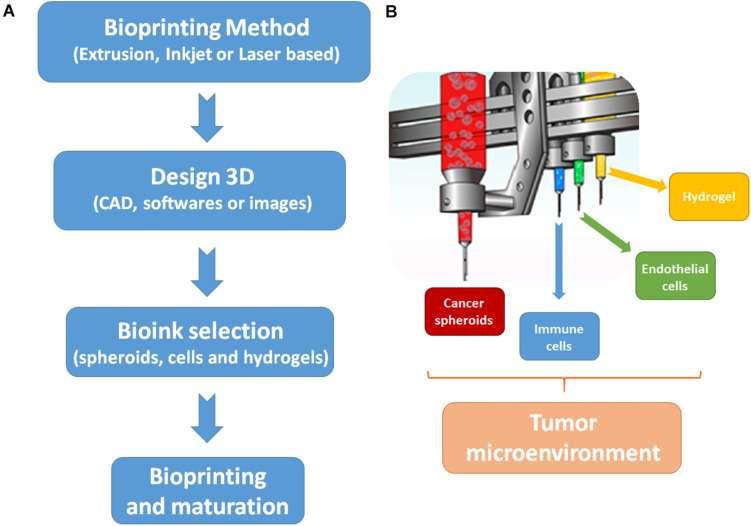
Steps and bioinks to biofabricate the tumor microenvironment by 3D bioprinting. **(A)** Steps to start the biofabrication process. First, it is necessary to choose a bioprinting method, which will complement the desired output. The majority of studies to develop cancer models are done with extrusion-base techniques. Then, the 3D design of the cancer model must be made by software or can be based on images. Next, it is necessary to choose the biological (cells or spheroids) and biomaterial (usually hydrogels) components of the bioinks. Finally, the bioprinting process can be started and tissue maturation can be carried out post printing. **(B)** Bioinks to replicate tumor microenvironments. In order to mimic the tumor microenvironment, the main bioinks are tumor spheroids, immune cells, endothelial cells, and a hydrogel to support cells proliferation and survival.

For recreating the tumor microenvironment, there is a need of tumor ECM reconstitution and the recreation of tumor vasculature (Liu et al., 2020). In Figure 2, the essential steps and bioinks to recreate the tumor microenvironment by 3D bioprinting are proposed. The ECM of the tumor microenvironment is composed of different proteins and stromal cells, but it is known that the composition of tumor ECM is tumor and patient specific. In addition, the biomechanical properties of tumor ECM can regulate tumor behavior and progression (Zhang Y. S. et al., 2016).

Recently, a considerable number of studies were performed to develop tumor models by 3D bioprinting. Table 1 reviews some of these studies. Dai et al. (2017) focused on replicating tumor microenvironments by improving tumor and stromal cell interactions in 3D bioprinted constructs. Their strategy relied on the self-assembly of multicellular heterogeneous brain tumor cell fibers by extrusion-based bioprinting. These fibers were part of the tumor ECM of the brain tumor. The morphological results showed that the construct was viable, proliferative, and presented tumor-stromal cell interactions. Hermida et al. (2020) used extrusion-based bioprinting to engineer glioblastoma models made of cancer, microglia, and stromal cells bioprinted within alginate modified with RGDS cell adhesion peptides, hyaluronic acid, and type I collagen. The glioblastoma cells presented more resistance to chemotherapeutic drugs in 3D engineered bioprinted constructs compared with monolayer cultures.

**TABLE 1:** Biofabrication of cancer models by 3D bioprinting.

Authors and year	Aim	Bioprinting technique	Bioink	Main result	Article title and *journal*
Dai et al., 2017	Improve tumor and stromal cell interactions by the development of 3D bioprinted constructs	Extrusion	Cancer cells within alginate, gelatin and fibrin	The construct was viable and resembled properly tumor and stromal cell interactions found in the *in vivo* tumor	Coaxial 3D bioprinting of self-assembled multicellular heterogeneous tumor fibers. *Scientific reports.*
Hermida et al., 2020	Engineer a 3D construct of a glioblastoma model by bioprinting	Extrusion	Cancer, microglia and stromal cells within alginate/RGDS, hyaluronic acid and collagen I hydrogels	The biofabricated glioblastoma model was functional and showed resistance to drugs	Three dimensional *in vitro* models of cancer: Bioprinting multilineage glioblastoma models. *Advances in biological regulation.*
Jiang et al., 2017	3D bioprinting of cells to produce complex spheroids models	Extrusion	Cancer and fibroblasts cells within alginate/gelatin hydrogel	The biofabricated spheroids were viable and increased in size over time	Directing the Self-assembly of Tumor Spheroids by Bioprinting Cellular Heterogeneous Models within Alginate/Gelatin Hydrogels. *Scientific reports.*
Kingsley et al., 2019	Biofabrication of tumor organoids by 3D bioprinting	Laser-assisted	Decellularized rat and human breast tissue extracellular matrix	The hydrogel supported the biofabrication of breast cancer cell organoids	Laser-based 3D bioprinting for spatial and size control of tumor spheroids and embryoid bodies. *Acta Biomaterialia.*
Schmidt et al., 2019	Biofabrication of melanoma constructs	Extrusion	Melanoma cells within matrigel, two different types of commercially available bioinks, with or without RGD sequence/laminin-mixture	The melanoma cells were able to spread, proliferate and create networks in the hydrogels	Tumor Cells Develop Defined Cellular Phenotypes After 3D-Bioprinting in Different Bioinks. *Cells.*
Swaminathan et al., 2019	Biofabrication of breast cancer spheroids by 3D bioprinting	Extrusion	Breast epithelial cells and alginate	Spheroids were formed and resistant to drugs, replicating better the tumor microenviroment	Bioprinting of 3D breast epithelial spheroids for human cancer models. *Biofabrication.*
Maloney et al., 2020	Biofabrication of glioblastoma and sarcoma organoids by 3D bioprinting	FRESH	Cancer cells within hyaluronic acid and collagen	The organoids were biofabricated, presented a spherical shape and can be used for drug screening tests	Immersion Bioprinting of Tumor Organoids in Multi-Well Plates for Increasing Chemotherapy Screening Throughput. *Micromachines.*
Han et al., 2020	Recapitulate tumor microenviroment with spheroids by 3D bioprinting	Extrusion	Fibroblasts and endothelial cells in gelatine, alginate and fibrinogen	Microvessel sprouting in the construct, increase of spheroids size and efficacy in drug screening tests	3D Bioprinted Vascularized Tumor for Drug Testing. *International journal of molecular sciences.*

Despite the development described above for spheroid bioprinting strategies, several studies have shown the spontaneous formation of spheroids after 3D bioprinting, reaching the mimicry of specific cancer types. Jiang et al. (2017) developed a proof of concept study by bioprinting a cross-linked alginate/gelatin hydrogel composed of breast cancer lineage cells and fibroblasts. After 1 week in culture, breast cancer cells formed viable spheroids that increased in size over time and attracted migrating fibroblasts through a matrix region of the hydrogel, which infiltrated the breast cancer spheroids.

Using the technique of 3D bioprinting named “laser direct write,” Kingsley et al. (2019) used microbeads to allow the formation and growth of multicellular tumor spheroids with homogeneous size and shape. The decellularized rat and human breast tissue ECM was used as a bioink for organoid formation by 3D bioprinting (Mollica et al., 2019). The principal advantage in this strategy is that these ECM hydrogels keep the structural and signaling cues of the breast cancer environment, which can determine a cell’s fate. The results showed that the hydrogel supported the production of breast cancer cell organoids allowing their use to engineer more complex organoids models to pre-clinical assays.

Schmidt et al. (2019) compared the interaction of different bioprinted hydrogels with melanoma cells. In total, five hydrogels were tested: matrigel and two different types of commercially available bioinks, with or without RGD sequence/laminin mixture. In Matrigel, melanoma cells were able to spread, proliferate, and produce networks in the construct, while in gelatin methacrylate melanoma cells grow in clusters. As expected, the choice of the bioink is crucial for the behavior of cancer cells in engineered constructs.

Human breast epithelial cell lines can be bioprinted as a cell suspension or as formed spheroids in alginate-based bioinks. These cells only formed spheroids in Matrigel-based biolinks and pre-formed spheroids kept their morphology and viability after bioprinting. When spheroids were formed, breast cancer cells were more resistant to drug assessment, replicating the tumor microenvironment (Swaminathan et al., 2019).

Maloney et al. (2020) used an immersion printing technique approach to perform the 3D bioprinting of tissue organoids in 96-well plates. The results showed that the bioink allowed the maintenance of the organoid structure. In the study, the bioink was composed of hyaluronic acid and collagen and was printed in a support bath made of gelatin. This innovative strategy, named as “Freeform Reversible Embedding of Suspended Hydrogels” (FRESH) is being largely explored to bioprint soft tissues without a scaffold, because it allows the maintenance of the biological structure after the removal of the support bath. To the best of our knowledge, this is the only technique at the moment that can be used to bioprint organoids. More importantly, the authors proved with patient-derived glioblastoma and sarcoma organoids that it is possible to use the method for drug screening tests *in vitro*.

Han et al. (2020) used 3D bioprinting to recapitulate the tumor microenvironment using spheroids. The method consisted of the biofabrication of a blood vessel layer engineered by fibroblasts and endothelial cells in gelatin, alginate, and fibrinogen, followed by the seeding of multicellular tumor spheroids of glioblastoma cells onto this blood vessel layer. The main results showed the sprouting of blood vessels with an increase in spheroid size. In addition, drug testing was performed and the biofabricated construct was sensitive to the treatment, showing that it can be used for drug efficacy tests *in vitro*.

However, there is an important limitation of these models that use hydrogels for drug testing. HTS analysis based on luminescence/fluorescence cannot be applied to these models due to the presence of hydrogels which are high viscous biomaterials. Another issue related to hydrogels is the small volume used in some applications because it can impair HTS tests (Yu et al., 2018). Table 2 summarizes the main characteristics, advantages, and disadvantages of 3D models described in this review.

**TABLE 2:** Current 3D models for recapitulating tumor microenvironment.

3D models	Materials for 3D models preparation	Cell types	Mimicry level
Spheroids	• Plates and rotors for cultivation such as spinner flasks, rotary cell culture systems, liquid overlay, micropatterned plates, low binding plates, microfluidics device • Culture in the presence or absence of fetal bovine serum (FBS)	• Can be obtained from cancer stem cell (CSC) population, tissue-derived tumor spheres such as lung, bladder, prostate, or breast cancer tissue and uveal melanoma, including cell lineages • Some tumor cells form spheres spontaneously, while others require additional manipulations	+
Organoids	• Cultured on diverse matrices such as Matrigel, collagen type I, HA (hyaluronic acid) hydrogel, PEG hydrogel, fibrin/laminin hydrogel	• Can be obtained from tumor cells isolated from tumor tissue such as metastatic colorectal carcinoma tissue, cervical carcinoma biopsy tissue, tumors of the gastrointestinal tract, prostate tumor cell lines • Can also be obtained from non-tumor organoids using gene-editing techniques	++
Bioprinting	• The 3D computer models containing information such as complex 3D geometries surface information can be created using MRI or CT scans • Bioreactors for tissue maturation in post-processing	• Multiple types of cancer cells including primary cancer cells, circulating tumor cells, and stromal cells including fibroblasts, endothelial cells and stem cells can be used for printing personalized tumor construct	+++

**3D models**	**Advantages**	**Disadvantages**

Spheroids	• Presence of gas, nutrient and pH gradients • Co-culture • Cultures without expensive cultivation methods • Reproduction of cell-cell and cell-ECM interactions • The screening of personalized drug can be performed with very small quantities of chemotherapeutic candidates	• Gradient structure complicates drug testing • Fragile structure • Difficulty of forming homogeneous spheroids • Cannot completely recapitulate the cellular and microenvironmental heterogeneity of physiological tumor tissue
Organoids	• Reproduction of cell-cell and cell-ECM interactions • Co-culture Primary tumor cells • Long-term cultivation • Stable at passaging	• Gradient of gases, nutrients and pH is not always reproducible • Therapeutic responses may depend on the matrix • High cost method
Bioprinting	• Enables the generation of cell laden cancer tissue constructs that can recapitulate the features of various types of cancers • Expressed characteristics of *in vivo* tumor tissues, such as high growth rates of cancer cells, aggressive invasiveness, angiogenesis, metastasis, high resistance to anticancer drugs. • Can supplement animal xenograft models because they maintain cancer–stromal cell interactions. • Can integrate perfusable vascular networks, automation and high-throughput testing • The inkjet bioprinting have low cost, fast printing and widely accessible • Non-contact and high cell viability in the Laser-assisted bioprinting (LAB) • Deposition of high-density cells in the Extrusion bioprinting	• A single bioprinting method cannot yet produce synthetic tissues and organs at all scales and complexities. • The inkjet bioprinting has drawbacks in terms of material viscosity. • The microextrusion bioprinting may need materials having crosslinking mechanisms or shear reduction properties not to affect cell viability • The difficulty in developing well-established vascular network within tumors • Require a labor -intensive and high cost • The inkjet bioprinting may have Nozzle clogging • Complex operation and time consuming preparation in laser-assisted bioprinting (LAB) • Low cell viability in extrusion bioprinting

**3D models**	**Adaptable to the HTS system**	**References**

Spheroids	+++	Sutherland, 1988; Wartenberg et al., 2001; Del Duca et al., 2004; Mazzoleni et al., 2009; Hardelauf et al., 2011; Li et al., 2011; Vinci et al., 2012; Froehlich et al., 2018; Ruiz et al., 2019
Organoids	+	Lötjönen et al., 2010; Vries et al., 2015; Fujii et al., 2016; Nanki et al., 2018; Xu et al., 2018; Fan et al., 2019; Lin et al., 2019; Nunes et al., 2019; Fiorini et al., 2020
Bioprinting	N/A	Hopp et al., 2005; Koch et al., 2010; Gruene et al., 2011; Murphy and Atala, 2014; Orloff et al., 2014; Mandrycky et al., 2016; Zhu et al., 2016; Peng et al., 2017; Mohammadi and Rabbani, 2018; Datta et al., 2020; Emmermacher et al., 2020

*+, means less mimicry; ++, means intermediary mimicry; +++, higher mimicry.*

## Perspectives

Some studies already used 3D bioprinting to develop successful tumor models; however, to the best of our knowledge, the use of spheroids as a printable bioink to biofabricate tumor models has not been largely explored yet. As spheroids are a 3D model with complex cell-to-cell and cell-to-extracellular matrix interactions, it would be advantageous to use them as the main component of the bioink associated with the stromal components and immune cells. Tumor organoids show the main advantage of being derived from human cancer biopsies; however, their 3D bioprinting is still in its infancy due to their shape heterogeneity, lack of reproducibility, and complexity.

Furthermore, patient-derived 3D bioprinted tumor models could be successfully used for *in vitro* drug screening of anticancer drugs in large scale. However, some challenges need to be addressed before this step, especially related to the hydrogel composition. Some studies are already exploring how to optimize the hydrogel to not impair HTS tests and analysis (Barata et al., 2016; Sarkar and Kumar, 2016; Lee et al., 2018). In addition, ongoing studies are focusing on the development of combined microfluidic/bioprinted constructs to minimize the cost and facilitate HTS of a large number of cancer drugs for a particular patient in order to improve personalized medicine approaches (Augustine et al., 2021).

## Conclusion

3D bioprinting is a recent and innovative approach that offers the ability to create highly complex hierarchical 3D constructs with cells, biomaterials, and growth factors. Bioprinting methods have been developed and optimized in recent years in order to accurately replicate the morphology, functions, and physiology of a specific tissue and their *in vivo* microenvironment. As tumor microenvironments are complex in cell and extracellular matrix composition, 3D bioprinting holds great potential for applications in cancer research, in order to mimic more reliable tumor models and their vasculature (Knowlton et al., 2015; Albritton and Miller, 2017).

The use of 3D bioprinting can allow the positioning of tumor spheroids or organoids and the surrounding stromal and immune cells, commonly associated with this complex tumor microenvironment. The recapitulation of tumorigenesis will provide more reliable results to drug screening tests (Satpathy et al., 2018; Meng et al., 2019) and personalized medicine (Ma et al., 2018).

## Author Contributions

GK and LB contributed to conception and design of the manuscript. GK, GM, RT, and LB wrote the first draft of the manuscript. GK, GM, RT, and ÚK wrote sections of the manuscript. BM designed the table and performed the formatting of the manuscript. LB performed the main corrections and revised the manuscript. All the authors contributed to manuscript revision, read, and approved the submitted version.

## Conflict of Interest

The authors declare that the research was conducted in the absence of any commercial or financial relationships that could be construed as a potential conflict of interest.
